# Skin Toxicity as Predictor of Survival in Refractory Patients with *RAS* Wild-Type Metastatic Colorectal Cancer Treated with Cetuximab and Avelumab (CAVE) as Rechallenge Strategy

**DOI:** 10.3390/cancers13225715

**Published:** 2021-11-15

**Authors:** Davide Ciardiello, Vincenzo Famiglietti, Stefania Napolitano, Lucia Esposito, Nicola Normanno, Antonio Avallone, Tiziana Latiano, Evaristo Maiello, Filippo Pietrantonio, Chiara Cremolini, Giuseppe Santabarbara, Carmine Pinto, Teresa Troiani, Erika Martinelli, Fortunato Ciardiello, Giulia Martini

**Affiliations:** 1 Oncologia Medica, Dipartimento di Medicina di Precisione, Università degli Studi della Campania “L. Vanvitelli”, 80131 Napoli, Italy; davideciardiello@yahoo.it (D.C.); Vincenzo.famiglietti@yahoo.it (V.F.); stefania.napolitano@unicampania.it (S.N.); luciaesposito081@gmail.com (L.E.); teresa.troiani@unicampania.it (T.T.); erika.martinelli@unicampania.it (E.M.); fortunato.ciardiello@unicampania.it (F.C.); 2 Oncologia Medica, Fondazione IRCCS Casa Sollievo della Sofferenza, 71013 San Giovanni Rotondo, Italy; t.latiano@operapadrepio.it (T.L.); e.maiello@libero.it (E.M.); 3 Biologia Cellulare e Bioterapie, Istituto Nazionale per lo Studio e la Cura dei Tumori “Fondazione Giovanni Pascale”–IRCCS, 80144 Napoli, Italy; n.normanno@istitutotumori.na.it; 4 Oncologia Clinica Sperimentale Addome, Istituto Nazionale per lo Studio e la Cura dei Tumori “Fondazione Giovanni Pascale”–IRCCS, 80131 Napoli, Italy; a.avallone@istitutotumori.na.it; 5 Oncologia Medica, Fondazione IRCCS Istituto Nazionale dei Tumori, 20133 Milan, Italy; Filippo.pietrantonio@unimi.it; 6 Oncologia Medica, Azienda Ospedaliera Universitaria, Università di Pisa, 56121 Pisa, Italy; chiaracremolini@gmail.com; 7 Oncologia Medica, Azienda Ospedaliera di Rilievo Nazionale “S. G. Moscati”, 83100 Avellino, Italy; giuseppe.santabarbara@aornmoscati.it; 8 Medical Oncology Unit, Comprehensive Cancer Centre, AUSL-IRCCS di Reggio Emilia, 42122 Reggio Emilia, Italy; pinto.carmine@ausl.re.it

**Keywords:** rechallenge anti-EGFR, immunotherapy, skin toxicity, mCRC

## Abstract

**Simple Summary:**

Anti-EGFR-related skin toxicity has been described as a predictive biomarker of response in patients with RAS wild-type (WT) metastatic colorectal cancer (mCRC). With the CAVE mCRC trial we previously provided the first evidence of the activity of cetuximab plus avelumab as rechallenge treatment in pretreated chemo-refractory RAS WT mCRC. Nowadays, skin toxicity remains the only confirmed clinical biomarker of response to anti-EGFR treatment in mCRC. The role of skin toxicity has not yet been explored in a rechallenge setting. In this paper we provide a post-hoc analysis of the CAVE mCRC trial that investigated the role of skin toxicity as a predictive biomarker of activity of cetuximab plus avelumab treatment and its correlation with different clinico-molecular variables on survival at the univariate and multivariate levels. High-grade skin toxicity, together to the circulating tumor DNA RAS/BRAF/EGFR wild-type status were the only variables with an impact on PFS and OS.

**Abstract:**

The single-arm phase II CAVE mCRC trial evaluated the combination of cetuximab plus avelumab as rechallenge strategy in *RAS* wild-type (WT) metastatic colorectal cancer (mCRC) patients, with clinical response to first-line anti-EGFR-based chemotherapy, who progressed and received a subsequent line of therapy. The correlation of skin toxicity (ST) and different clinico-molecular variables with overall survival (OS), progression-free survival (PFS) and response rate (RR) was assessed at univariate and multivariate analysis. A total of 33/77 (42.9%) patients experienced grade 2–3 ST and displayed median OS (mOS) of 17.8 months (CI 95%, 14.9–20.6); whereas 44/77 (57.1%) patients with grade 0–1 ST exhibited mOS of 8.2 months (CI 95%, 5.5–10.9), (hazard ratio (HR), 0.51; CI 95%, 0.29–0.89; *p* = 0.019). Median PFS (mPFS) was 4.6 months (CI 95%, 3.4–5.7) in patients with grade 2–3 ST, compared to patients with grade 0–1 ST with mPFS of 3.4 months (CI 95%, 2.7–4.1; HR, 0.49; CI 95%, 0.3–0.8; *p* = 0.004). Grade 2–3 ST (HR, 0.51; CI 95%, 0.29–0.89; *p* = 0.019) and *RAS/BRAF/EGFR* WT circulating tumor DNA (ctDNA) (HR, 0.50; CI 95%, 0.27–0.9; *p* = 0.019) had a statistically significant effect on OS at univariate analysis. At the multivariate analysis, *RAS/BRAF/EGFR* WT ctDNA status maintained statistical significance (HR, 0.49; CI 95%, 0.27–0.9; *p* = 0.023), whereas there was a trend towards ST grade 2–3 (HR, 0.54; CI 95%, 0.29–1.01; *p* = 0.054). Skin toxicity is a promising biomarker to identify patients with mCRC that could benefit of anti-EGFR rechallenge.

## 1. Introduction

Different studies have shown promising activity of reintroduction of anti-epidermal growth factor receptor (EGFR) drugs in patients with *RAS* wild-type (*RAS* WT) metastatic colorectal cancer (mCRC), that obtained clinical benefit by first-line therapy with anti-EGFR drugs, then became resistant and progressed to second-line treatment [1,2,3,4,5,6]. This treatment strategy is called rechallenge. The biological rational relies on the arising of *RAS* mutant (*RAS* MT) cells during treatment with EGFR inhibitors (EGFRi), that lead to progression of disease (PD). During a subsequent EGFR-free therapeutic window, the acquired selected *RAS* MT clones progressively decay, while sensitive cells proliferate restoring vulnerability to EGFRi [7,8,9].

CAVE mCRC is a single-arm phase II study, in which 77 patients with refractory *RAS* WT mCRC received cetuximab plus the immune checkpoint inhibitor (ICI) avelumab, as rechallenge strategy [5]. In the intention-to-treat population (ITT population) the primary endpoint was met, with an improvement in median overall survival (mOS) [5]. The highest benefit was observed in patients with *RAS, BRAF,* and *EGFR* wild-type tumor assessed by basal plasma circulating tumor DNA (ctDNA) analysis [5].

So far, beside WT ctDNA at liquid biopsy analysis, several potential biomarkers have been investigated to predict the response to EGFRi rechallenge with discordant results [4,6,10].

The insurgence of skin toxicity (ST) is the most frequent adverse drug-related reaction (ADR) associated with EGFRi [11,12,13,14]. The physio-pathological mechanism of ST is due to the role of the EGFR pathway in the homeostasis of healthy tissues including skin and adnexal [11,12]. Therefore, EGFRi dysregulates keratinocyte proliferation, differentiation, migration, production of pro-inflammatory cytokines, and immune infiltration. Interestingly, strong evidence suggests a correlation between the appearance and intensity of ST and clinical outcome [15,16,17,18,19,20]. In mCRC robust data coming from large phase II/III clinical trials showed that patients treated with the monoclonal antibodies (mAbs) cetuximab and panitumumab had significant improvement in response rate (RR) and overall survival (OS) in case of insurgence of ST of grade 2 or higher [17,18,19,20]. In this scenario, in the absence of predictive biomarkers of response to rechallenge with EGFRi, we sought to address the impact of ST in terms of OS, PFS, and RR in the CAVE mCRC study.

## 2. Materials and Methods

### 2.1. Study Design and Patient Population

CAVE mCRC is a non-profit, single-arm, academic, open-label, phase II trial [5]. Patients enrolled in the study had: histologically confirmed mCRC with *RAS* (*NRAS* and *KRAS*, exon 2, 3 and 4) WT tumors, obtained a complete (CR) or partial response (PR) during a first-line treatment with an anti-EGFR-based regimen and should have progressed, and received at least one subsequent line of therapy with an interval of more than 4 months from last dose of the anti-EGFR drug. Additional inclusion and exclusion criteria are described in the full protocol available online [5].

This trial is registered with Eudract.ema.europa.eu, EudraCT number: 2017-004392-32 and ClinicalTrial.gov identifier: NCT04561336.

### 2.2. Patient Monitoring and Response Assessment

Clinical monitoring of patient safety was constantly assessed, and toxicity was graded using the National Cancer Institute-Common Toxicity Criteria (NCI-CTC) for adverse events, version 4.03. With the term skin toxicity, we included acneiform, erythematosus, maculo-papular rash, and cutaneous xerosis.

Tumor evaluation was assessed according to RECIST criteria, version 1.1, by using spiral or conventional CT scan, radiography, or MRI, if required. Tumor measurements were performed at baseline, and every 8 weeks for 40 weeks and every 12 weeks thereafter. Patients were followed until progression, regardless of whether study treatment was discontinued or delayed. Radiological results were evaluated by local investigators and confirmed by coordinating center investigators. All data, toxicities, and serious adverse events were collected in the electronic case report form. All patients provided written informed consent before entering the trial. The study was conducted in accordance with the principles of the Declaration of Helsinki and the International Conference on Harmonization and Good Clinical Practice guidelines.

### 2.3. qPCR Analysis of Plasma Samples

Plasma specimens of 67 out of 77 patients were collected at baseline and were suitable for ctDNA evaluation of *KRAS, NRAS, BRAF,* and *EGFR* extracellular domain S492R mutations by using the automated Idylla ^TM^ qPCR-based platform.

The results of the analyses were visualized using the on-line tool Idylla ^TM^ Explore (idyllaexplore.biocartis.com, last access 30 May 2020). The protocol has been previously validated and is fully described elsewhere [21].

### 2.4. Statistical Analysis

Several clinical factors were evaluated for their correlation with median mPFS, mOS, and overall response rate (ORR). PFS and OS were calculated using the Kaplan–Meier method. The chi-square and Fisher tests were used to assess the correlation between clinical variables and PFS, OS, and ORR at univariate and multivariate analysis. Statistical analyses were performed using the SPSS package (v.23).

## 3. Results

Between August 2018 and February 2020, 77 patients were enrolled in CAVE mCRC trial and received the combination of avelumab plus cetuximab as rechallenge therapy. The main patient characteristics are listed in Table S1. ITT population survival outcomes have been previously described [5].

**Figure 1:**
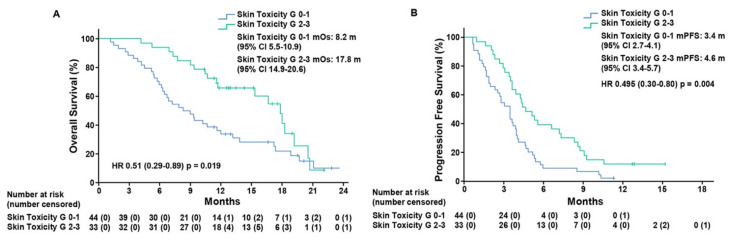
Kaplan–Meier estimates of overall survival (**A**) and progression-free survival (**B**) in the intention-to-treat population according to skin toxicity grade 0–1 and grade 2–3. Grade, G; median overall survival, mOS; median progression-free survival, mPFS; months, m.

To assess the predictive value of ST as a biomarker of response to treatment, we performed a post-hoc analysis of patients that was based on the grading of skin toxicity. Thirty-three (42.9%) patients, that experienced grade 2–3 ST, presented mOS of 17.8 months (CI 95%, 14.9–20.6) as compared to 44 (57.1%) patients with grade 0–1 ST, who displayed mOS of 8.2 months (CI 95%, 5.5–10.9), (hazard ratio (HR), 0.51; CI 95%, 0.29–0.89; *p* = 0.019)). mPFS was 4.6 months (CI 95%, 3.4–5.7) in patients with grade 2–3 ST as compared to patients with grade 0–1 ST, who exhibited mPFS of 3.4 months (CI 95%, 2.7–4.1), (HR, 0.49; CI 95%, 0.3–0.8; *p* = 0.004) (Figure 1).

To further investigate the impact of skin toxicity on patient outcome, we did univariate and multivariate analyses with different clinical variables. At univariate analysis for PFS, besides skin toxicity, another three variables were associated with an improvement in PFS: number of metastatic sites ≤2 (HR, 0.54; CI 95%, 0.33–0.87; *p* = 0.013); surgery of primary tumor (HR, 0.58; CI 95%, 0.36–0.94; *p* = 0.028); and *RAS/BRAF/EGFR* WT ctDNA status at baseline liquid biopsy analysis (HR, 0,41; CI 95%, 0.23–0.75; *p* = 0.004). However, at multivariate analysis for PFS none of the analyzed parameters retained statistical significance (Table 1). Interestingly, grade 2–3 ST (HR, 0.51; CI 95%, 0.29–0.89; *p* = 0.019) and *RAS/BRAF/EGFR* WT ctDNA (HR, 0.50; CI 95%, 0.27–0.9; *p* = 0.019) were the only variables that had a statistically significant effect on OS at univariate analysis (Table 2). Of note, at the multivariate analysis *RAS/BRAF/EGFR* WT ctDNA status maintained statistical significance (HR, 0.49; CI 95%, 0.27–0.9; *p* = 0.023), whereas there was a trend towards ST grade 2–3 (HR, 0.54; CI 95%, 0.29–1.01; *p* = 0.054).

**Figure 2:**
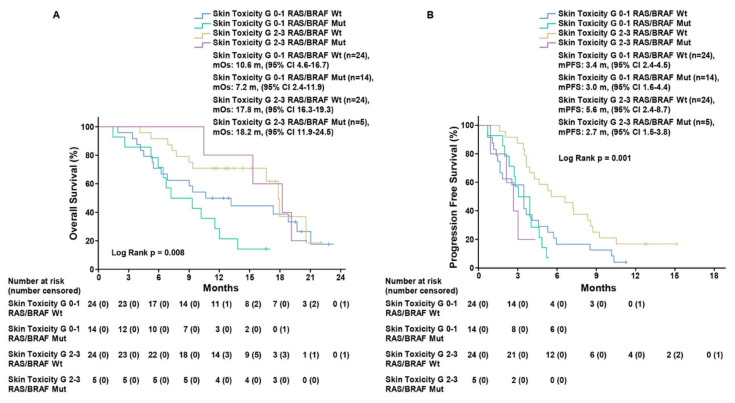
Kaplan–Meier estimates of overall survival (**A**) and progression-free survival (**B**) in patients with RAS/BRAF/EGFR wild-type and mutant circulating DNA according to skin toxicity grade 0–1 and grade 2–3. Grade, G; median overall survival, mOS; median progression-free survival, mPFS; months, m.

We also evaluated RR and disease-control rate (DCR) in each defined subgroup (Table 3). In the 33 patients with grade 2–3 ST, 1 (3%) CR, 2 (6.1%) PR, and 24 (72.7%) SD were observed, with DCR of 81.8%. On the other hand, in the 44 patients with grade 0–1 ST, we found 0 CR, 3 (6.8%) PR, 20 (45.5%) SD, with 52.3% DCR. To date, the only recognized clinical biomarker of response to rechallenge strategy with anti-EGFR drugs is represented by the absence of mutations in *RAS/BRAF/EGFR* genes [2,3,5,6]. Therefore, we assessed the impact of ST in the 67 patients with basal *RAS/BRAF/EGFR* ctDNA analysis, divided into four subgroups: *RAS/BRAF/EGFR* WT ST 2–3, *RAS/BRAF/EGFR* WT ST 0–1, *RAS/BRAF/EGFR* mutant ST 2–3, and *RAS/BRAF/EGFR* mutant ST 0–1. Kaplan–Meier analysis showed that mOS was 17.8 months (CI 95%, 16.3–19.3) in the 24 patients with *RAS/BRAF/EGFR* WT ctDNA at baseline with grade 2–3 ST as compared to mOS of 10.6 months (CI 95%, 4.6–16.6), (HR, 0.64; CI 95%, 0.30–1.39; *p* = 0.26) for those 24 patients with grade 0–1 ST. Regarding the subgroup of 19 patients with *RAS/BRAF/EGFR* mutant ctDNA at baseline, mOS was 18.2 months (CI 95%, 11.9–24.5) for 5 patients that experienced grade 2–3 ST as compared with 7.2 months (CI 95% 2.4–11.9), (HR, 0.17; CI 95%, 0.038–0.81; *p* = 0.026) for 14 patients with grade 0–1 ST (log-rank *p* = 0.001) (Figure 2, Figure S1 and Figure S2). mPFS was 5.6 months (CI 95%, 2.4–8.7) in *RAS/BRAF/EGFR* WT patients with grade 2–3 ST as compared to patients with grade 0–1 ST, who exhibited mPFS of 3.4 months (CI 95%, 2.4–4.5), (HR, 0.49; CI 95%, 0.26–0.9; *p* = 0.021). For the 19 patients with, *RAS/BRAF/EGFR* mutant tumor, mPFS was 2.7 months (CI 95% 1.5–3.8) for 5 patients with grade 2–3 ST and 3.0 months (CI 95% 1.6–4.4) for 14 patients with grade 0–1 ST (HR, 1.93; CI 95%, 0.65–5.72; *p* = 0.23) (log-rank *p* = 0.008) (Figure 2, Figure S1 and Figure S2).

## 4. Discussion

Dermatologic toxicities such as acneiform rash, dry skin, pruritus, erythema, and paronychia have been reported in more than 90% patients receiving anti-EGFR monoclonal antibodies for the treatment of mCRC, although the majority of these toxicities are of mild grade [22]. Acneiform rash is the most frequent dermatologic ADR that could negatively impact treatment outcome, not only due to the risk of more intense adverse events such as infections and pruritus, which could lead to dose modifications and interruptions, but also because of the psychological impact on patient everyday life [11]. The correlation between EGFR blockade-related ST and response to treatment has been largely investigated in phase II and phase III clinical trials and, to date, ST represents the only clinical marker of response to cetuximab or panitumumab treatment [17]. Conversely, the EVEREST trial, which aimed to consider dose escalation of cetuximab in patients experiencing no skin toxicity after 21 days of treatment with the scope of achieving better outcomes, showed that the increase in grade 2 ST after cetuximab dose intensification did not correlate with improved OS. Interestingly, RR was higher only in patients with *KRAS* WT tumors [23]. Recently, a post-hoc analysis of the FIRE3 trial showed that the occurrence of grade 2 ST and of early tumor shrinkage were the only factors associated with improved survival in mCRC *RAS* WT patients treated with chemotherapy plus cetuximab compared with bevacizumab [20].

Except for an analysis conducted by Santini and colleagues, which reported a significant correlation between ST experienced during first-line cetuximab treatment and irinotecan plus cetuximab rechallenge in *KRAS* WT patients, the potential predictive role of ST that occurs during rechallenge treatment has not been investigated [1]. In the CAVE mCRC trial ST was a common adverse event that was related to cetuximab treatment with 33/77 patients having grade 2–3 ST and 44/77 presenting grade 0–1 ST. The occurrence of grade 2–3 ST is a strong predictor of patient outcome. In fact, in the ITT population, grade 2–3 versus grade 0–1 ST was correlated with an improvement in OS (HR, 0.51; CI 95%, 0.29–0.89; *p* = 0.019) and in PFS (HR, 0.49; CI 95%, 0.3–0.8; *p* = 0.004).

Different clinical factors have been investigated to identify the population that could really benefit from anti-EGFR rechallenge. So far, the absence of resistance mutations at liquid biopsy analysis is the only available biomarker to predict response to EGFRi rechallenge. Interestingly, in the CAVE mCRC trial, ST and *RAS/BRAF/EGFR* WT ctDNA were the only two parameters associated with a statistically significant increase in overall survival at univariate analysis. Multivariate analysis confirmed the value of *RAS/BRAF/EGFR* WT ctDNA status, with a trend towards statistical significance for skin toxicity. Moreover, patients with grade 2–3 ST had DCR of 81.8% as compared to 52.3% in the subgroup with grade 0–1 ST, with only six patients experiencing PD at first evaluation. These are very promising data that have to be put in the context of a chemorefractory mCRC disease setting, in which responses to regorafenib and trifluoridin/tipiracil are relatively rare and DCR is approximately 40% [24,25].

Of note, when comparing survival outcomes of the four defined subgroups: *RAS/BRAF/EGFR* WT ST 2-3, *RAS/BRAF/EGFR* WT ST 0–1, *RAS/BRAF/EGFR* mutant ST 2-3, and *RAS/BRAF/EGFR* mutant ST 0–1, even for patients with *RAS/BRAF/EGFR* WT tumors at basal plasma ctDNA analysis the highest benefit was observed in those that experienced grade 2–3 ST, with improvement in mPFS of approximately 2 months (5.6 months) compared with the other three subgroups. On the other hand, regarding OS, when experiencing a high-grade ST, patients with *RAS/BRAF/EGFR* mutant tumors present similar outcomes (18.2 months) with *RAS/BRAF/EGFR* WT (17.8 months), vs. 10.6 months and 7.2 months in patients experiencing grade 0–1 ST with *RAS/BRAF/EGFR* WT and mutant tumors, respectively. Considering the efficacy of treatment in *RAS/BRAF/EGFR-*mutated patients we could speculate on the contribution of avelumab to an anti-EGFR treatment as rechallenge strategy. In fact, we know that the combination of an anti-EGFR and an anti-PDL1 antibody could enhance the ADCC and therefore providing better efficacy that a single agent anti-EGFR drug. However, the data show a discrepancy between OS and PFS results, with a clear advantage in OS for patients with *RAS/BRAF/EGFR* mutant experiencing a high-grade ST that is not reflected in PFS, which remains poor and comparable to the subgroups of patients with G0–1 skin toxicity, independently from the *RAS/BRAF/EGFR* WT or mutant status. Subsequent lines of treatment and the small sample of *RAS/BRAF/EGFR* mutant patients (5 patients with G2-3 ST, 14 patients with G0–1 ST) could have affected the PFS results. Thus, the nature of our study (single-arm, small sample of patients) limits us in solving the doubt about OS-PFS discrepancy.

The CAVE mCRC represents, so far, the first evidence of the activity of an anti-EGFR monoclonal antibody in combination with an immune checkpoint inhibitor as rechallenge treatment in pretreated chemo-refractory RAS WT mCRC. The rational of the potential effectiveness of this novel combination derives from the induction of antibody-dependent cell-mediated cytotoxicity (ADCC) in enhancing antitumor activity from the IgG1 iso-type monoclonal antibodies avelumab and cetuximab [26,27,28]. In this respect, preclinical and clinical data from our group demonstrated how this combination increased ADCC in human non-small-cell lung cancer (NCSLC) cell lines and determined NK-cell-driven ADCC in chemo-refractory NSCLC patients in a proof-of-concept study [29].

Acneiform rash is a result of EGFRi at skin level, which could be related to the recruitment of immune cells and the consequent inflammatory response [30,31]. Specifically, blocking EGFR with mAbs leads to the release of type I interferon (IFN) in keratinocytes, which causes improved antigen presentation in the tumor microenvironment with T cell recruitment. On the other hand, IFN modulates immune response by activating cytokines and chemokines (TNF alpha, IL-1 Beta, IL-6, and IL-10) [32]. All these mechanisms could be the effectors of ST, with loss of antimicrobial response and damage to the epithelial barrier. Our study has different limitations. Due to the single-arm design it is very difficult to discern the impact of avelumab in the development of skin toxicity. Although cetuximab skin toxicity, with acneiform rash being the principal clinical manifestation, has been extensively described in mCRC, there is poor evidence regarding anti PD-L1 immune-related skin toxicity in the same setting, considering that, unfortunately, only a few patients with an MSI tumor (almost 5%) could really benefit from CPI treatment [17,18,19,20,21,26]. However, immune-related skin toxicity is well described in other solid tumors and has been reported with a wide range of clinical manifestations. In the CAVE mCRC trial, the principal clinical manifestation of skin toxicity that has been reported is an acneiform rash, that we have correlated with anti-EGFR activity. Moreover, the onset of cutaneous toxicity has been between cycle 2 and cycle 3 for most of our patients (75 out of 77), while CPI-related dermatologic adverse events seem to have a delayed onset. Future randomized analyses with a cetuximab single-agent control arm will clarify how avelumab contributes to ST.

## 5. Conclusions

With the limitations of the single-arm non-randomized design of the trial, the CAVE mCRC study demonstrates a potential role of ST as predictive biomarker of response to anti-EGFR retreatment. In the future, the implementation of translational analyses to be integrated with plasma ctDNA analysis in the context of a randomized and larger trial is necessary to confirm the potential role of ST as a surrogate of response.

## Figures and Tables

**Table 1:** Association of clinical variables with median progression-free survival by univariate and multivariate analysis.

Univariate Analysis				Multivariate Analysis	
Variable	Number of Patients	Hazard Ratio (CI 95%)	*p*-Value	Hazard Ratio (CI 95%)	*p*-Value
Skin Rash					
Grade 0–1	44 (57.1%)	1.00 (ref)		1.00 (ref)	
Grade 2–3	33 (42.9%)	0.495 (0.30–0.80)	0.004	0.71 (0.39–1.27)	0.25
Sex					
Female	35 (45.5%)	0.89 (0.56–1.42)	0.64		
Male	42 (54.5%)	1.00 (ref)			
ECOG					
0	52 (67.5%)	0.78 (0.48–1.31)	0.37		
1	25 (32.5%)	1.00 (ref)			
Lines of treatment					
III	56 (72.7%)	1.00 (ref)			
>III	21 (27.3%)	0.87 (0.51–1.47)	0.61		
Number of metastatic sites					
≤II	45 (58.4%)	0.54 (0.33–0.87)	0.013	0.72 (0.41–1.25)	0.24
>II	32 (41.6%)	1.00 (ref)		1.00 (ref)	
Surgery of the primary tumor					
Yes	48 (62.3%)	0.58 (0.36–0.94)	0.028	0.70 (0.41–1.20)	0.20
No	29 (37.7%)	1.00 (ref)		1.00 (ref)	
Microsatellites Instability					
MSI	3 (5.41%)	0.59 (0.18–1.91)	0.59		
MSS	71 (95.9%)	1.00 (ref)			
Sidedness					
Left and rectum	72 (93.5%)	0.45 (0.17–1.15)	0.096		
Right	5 (6.5%)	1.00 (ref)			
Synchronous metastases					
Yes	56 (72.7%)	1.00 (ref)			
No	21 (27.3%)	0.64 (0.37–1.10)	0.11		
RAS/BRAF/EGFR mutational status					
WT	48 (71.6%)	0.41 (0.23–0.75)	0.004	0.58 (0.3–1.08)	0.10
MT	19 (28.4%)	1.00 (ref)		1.00 (ref)	

CI, confidence intervals; Eastern Cooperative Oncology Group performance status, ECOG; microsatellite instability, MSI; microsatellite-stable, MSS; wild-type, WT; mutant, MT.

**Table 2:** Association of clinical variables with median overall survival by univariate and multivariate analysis.

Univariate Analysis				Multivariate Analysis	
Variable	Number of Patients	Hazard Ratio (CI 95%)	*p*-Value	Hazard Ratio (CI 95%)	*p*-Value
Skin Rash					
Grade 0–1	44 (57.1%)	1.00 (ref)		1.00 (ref)	
Grade 2–3	33 (42.9%)	0.51 (0.29–0.89)	0.019	0.54 (0.29–0.1.01)	0.054
Sex					
Female	35 (45.5%)	1.03 (0.60–1.76)	0.90		
Male	42 (54.5%)	1.00 (ref)			
ECOG					
0	52 (67.5%)	0.64 (0.36–1.11)	0.11		
1	25 (32.5%)	1.00 (ref)			
Lines of treatment					
III	56 (72.7%)	1.00 (ref)			
>III	21 (27.3%)	1.12 (0.63–1.99)	0.69		
Number of metastatic sites					
≤II	45 (58.4%)	0.74 (0.43–1.27)	0.28		
>II	32 (41.6%)	1.00 (ref)			
Surgery of the primary tumor					
Yes	48 (62.3%)	0.65 (0.38–1.11)	0.12		
No	29 (37.7%)	1.00 (ref)			
Microsatellites Instability					
MSI	3 (5.41%)	1.25 (0.30–5.1)	0.75		
MSS	71 (95.9%)	1.00 (ref)			
Sidedness					
Left and rectum	72 (93.5%)	1.25 (0.44–3.47)	0.67		
Right	5 (6.5%)	1.00 (ref)			
Synchronous metastases					
Yes	56 (72.7%)	1.00 (ref)			
No	21 (27.3%)	0.73 (0.39–1.37)	0.34		
RAS/BRAF/EGFR mutational status					
WT	48 (71.6%)	0.50 (0.27–0.90)	0.022	0.49 (0.27–0.90)	0.023
MT	19 (28.4%)	1.00 (ref)		1.00 (ref)	

CI, confidence intervals; Eastern Cooperative Oncology Group performance status, ECOG; microsatellite instability, MSI; microsatellite-stable, MSS; wild-type, WT; mutant, MT.

**Table 3:** Association of clinical variables with overall response rate.

Variable	Number of Patients	CR	PR	SD	PD
Skin toxicity					
Grade 0–1	44 (57.1%)	0 (0%)	3 (6.8%)	20 (45.5%)	21 (47.7%)
Grade 2–3	33 (42.9%)	1 (3%)	2 (6.1%)	24 (72.7%)	6 (18.2%)
Sex					
Female	35 (45.5%)	1 (2.9%)	3 (8.6%)	21 (60%)	10 (28.6%)
Male	42 (54.5%)	0 (0%)	2 (4.8%)	23 (54.8%)	17 (40.5%)
ECOG					
0	52 (67.5%)	1 (1.9%)	4 (7.7%)	29 (55.8%)	18 (34.6%)
1	25 (32.5%)	0 (0%)	1 (4.0%)	15 (60%)	9 (36.0%)
Lines of treatment					
III	56 (72.7%)	1 (1.8%)	3 (5.4%)	31 (55.4%)	21 (37.5%)
>III	21 (27.3%)	0 (0%)	2 (9.5%)	13 (61.9%)	6 (28.6%)
Number of metastatic sites					
≤II	45 (58.4%)	1 (2.2%)	2 (4.4%)	29 (64.4%)	13 (28.9%)
>II	32 (41.6%)	0 (0%)	3 (9.4%)	15 (46.9%)	14 (43.8)
Surgery of the primary tumor					
Yes	48 (62.3%)	1 (2.1%)	4 (8.3%)	31 (64.6%)	12 (25%)
No	29 (37.7%)	0 (0%)	1 (3.4%)	13 (44.8%)	15 (51.7%)
Microsatellites Instability					
MSI	3 (5.41%)	0 (0%)	0 (0%)	3 (100%)	0 (0%)
MSS	71 (95.9%)	1 (1.4%)	5 (7%)	40 (56.3%)	25 (35.2%)
Sidedness					
Left and rectum	72 (93.5%)	1 (1.4%)	4 (5.6%)	43 (59.7%)	24 (33.3%)
Right	5 (6.5%)	0 (0%)	1 (20%)	1 (20%)	3 (60%)
Synchronous metastases					
Yes	56 (72.7%)	1 (1.8%)	3 (5.4%)	30 (53.6%)	22 (39.3%)
No	21 (27.3%)	0 (0%)	2 (9.5%)	14 (66.7%)	5 (23.8%)
RAS/BRAF/EGFR mutational status					
WT	48 (71.6%)	1 (2.1%)	3 (6.3%)	31 (64.6%)	13 (27.1%)
MT	19 (28.4%)	0 (0%)	1 (5.3%)	8 (42.1%)	10 (52.6%)

Complete response, CR; partial response, PR; stable disease, SD; progression disease, PD; Eastern Cooperative Oncology Group performance status, ECOG; microsatellite instability, MSI; microsatellite-stable, MSS; wild-type, WT; mutant, MT.

## Data Availability

The data presented in this study are available on request from the corresponding author.

## Supplementary Materials

The followings are available online at https://www.mdpi.com/article/10.3390/cancers13225715/s1, Table S1: Baseline characteristics, Figure S1: Kaplan–Meier estimates of overall survival (A) and progression-free survival (B) in patients with RAS/BRAF/EGFR wild-type circulating DNA according to skin toxicity grade 0–1 and grade 2–3, Figure S2: Kaplan–Meier estimates of overall survival (A) and progression-free survival (B) in patients with RAS/BRAF/EGFR mutant circulating DNA according to skin toxicity grade 0–1 and grade 2–3.
