# Supplementary material for: Skin Toxicity as Predictor of Survival in Refractory Patients with RAS Wild-Type Metastatic Colorectal Cancer Treated with Cetuximab and Avelumab (CAVE) as Rechallenge Strategy
Source: Cancers (Basel). 2021 Nov 15;13(22):5715. doi: 10.3390/cancers13225715 (PMC8616320; doi:10.3390/cancers13225715)
Supplement: Supplementary file 1 [file cancers-13-05715-s001.zip › cancers-1447168-supplementary.pdf]

Supplementary Materials: Skin toxicity as predictor of survival in refractory patients with *RAS* wild type metastatic colorectal cancer treated with cetuximab and avelumab (CAVE) as rechallenge strategy

Davide Ciardiello, Vincenzo Famiglietti, Stefania Napolitano, Lucia Esposito, Nicola Normanno, Antonio Avallone, Tiziana Latiano, Evaristo Maiello, Filippo Pietrantonio, Chiara Cremolini, Giuseppe Santabarbara, Carmine Pinto, Teresa Troiani, Erika Martinelli, Fortunato Ciardiello and Giulia Martini

Table S1. Baseline characteristics.

	Patients (n°77)
**Sex**	
**Female**	35 (45.5%)
**Male**	42 (54.5%)
**ECOG**	
0	52 (67.5%)
1	25 (32.5%)
**Line of treatment**	
III	56 (72.7%)
>III	21 (27.3%)
**Number of metastatic sites**	
≤II	45(58.4%)
>II	32(41.6%)
**Surgery of the primary tumor**	
Yes	48 (62.3%)
No	29 (37.7%)
**Microsatellites Instability**	
MSI	3(5.41%)
MSS	71(95.9%)
**Sidedness**	
Left and rectum	72(93.5%)
Right	5 (6.5%)
**Synchronous metastases**	
Yes	56(72.7%)
No	21(27.3%)
**RAS/BRAF/EGFR mutational status**	
WT	48 (71.6%)
MT	19 (28.4%)
**Skin Rash**	
Grade 0-1	44 (57.1%)
Grade 2-3	33 (42.9%)

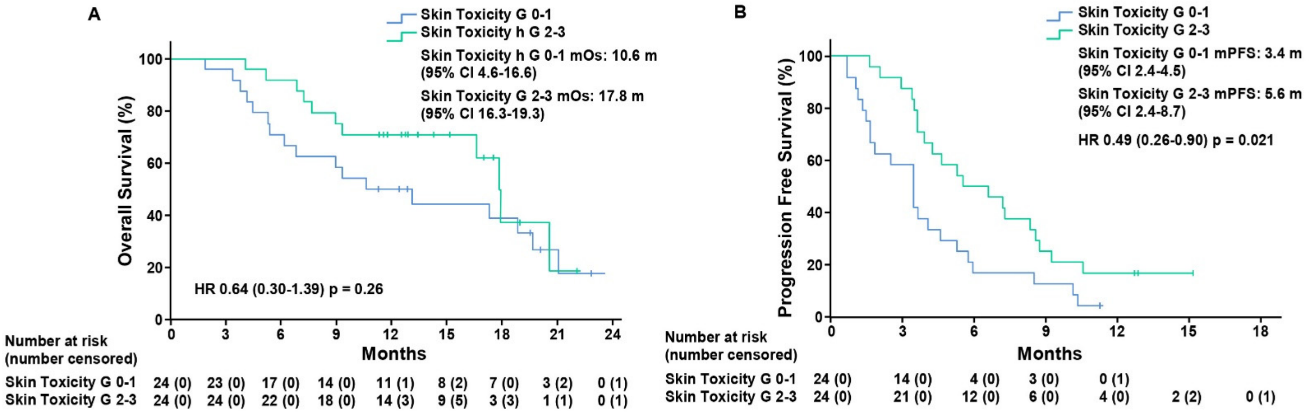

Figure S1. Kaplan–Meier estimates of overall survival (A) and progression-free survival (B) in patients with RAS/BRAF/EGFR wild-type circulating DNA according to skin toxicity grade 0–1 and grade 2–3.

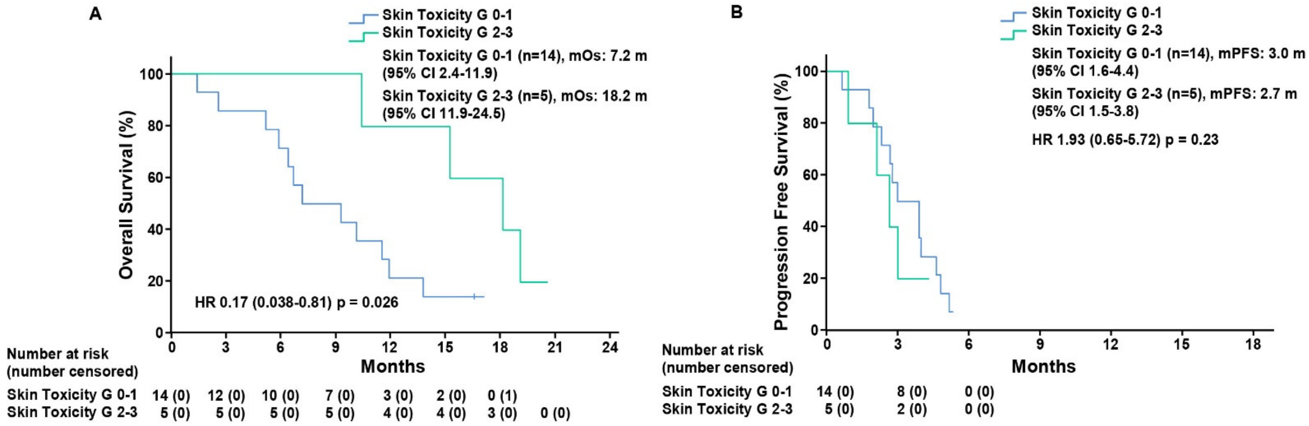

Figure S2. Kaplan–Meier estimates of overall survival (A) and progression-free survival (B) in patients with RAS/BRAF/EGFR mutant circulating DNA according to skin toxicity grade 0–1 and grade 2–3.
